# Aneurysmal dilatation of the pulmonary artery trunk and its major branches

**DOI:** 10.11604/pamj.2015.21.281.7219

**Published:** 2015-08-13

**Authors:** Ahmed Belkouch, Abdelilah Mouhsine

**Affiliations:** 1 Emergency Department, Mohamed V Military Hospital of Instruction, Hay Ryad Avenue des Far, Rabat 10100, Morocco; 2 Department of Radiology, Avicenna Military Hospital, Avenue Al Mouqaouama, Marrakech 40000, Morocco

**Keywords:** Aneurysmal dilatation, pulmonary artery, mitral valve stenosis

## Image in medicine

A 34-year-old woman with history of post rheumatic mitral stenosis since the age of 12 years at the stage of surgery (the patient refused it). She was admitted for cough and dyspnea of acute onset without fever or chest pain. The patient was in respiratory distress, afebrile and hemodynamically stable. Arterial blood gas analysis showed PaO2 = 48mmHg, PaCO2 = 56mmHg and pH = 7.26. Clinical examination found signs related to mitral stenosis and congestive heart failure. The electrocardiogram showed atrial fibrillation. Chest radiography showed no signs of pneumonia but highlighted cardiomegaly with a prominent left median arc and ectasia of the lower right arc (A). Doppler echocardiography showed a tight mitral stenosis with mitral area of 0.6 cm2 and a dilated left atrium and right cardiac cavities. It also revealed a significant tricuspid regurgitation and a major pulmonary hypertension with a systolic pulmonary artery pressure at 97 mmHg. The chest CT objectified aneurysmal dilatation of the pulmonary artery (8.2 cm) and its branches without signs of pulmonary embolism (B). The patient improved after non-invasive ventilation and diuretic treatment. Proximal pulmonary artery aneurysms, defined by a ratio of pulmonary artery to aortic diameter greater than two, are rare and bilateral aneurysms were exceptionally described. The causes are dominated by infectious diseases, inflammatory arteritis, and congenital heart disease and acquired valvular heart disease. Pulmonary arterial hypertension can cause chronic aneurysm of the pulmonary artery by direct infringement of the wall with atherosclerosis, medianecrosis and aneurysmal distension.

**Figure 1:**
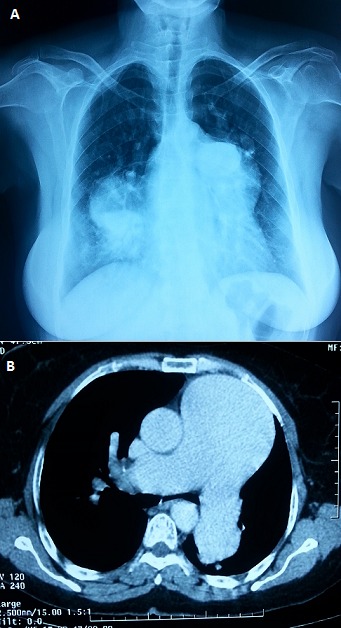
(A) chest radiography showing cardiomegaly and the pulmonary artery aneurysm; (B) CT scan showing the dilatation of the pulmonary artery trunk and its major branches. A ratio of pulmonary artery to aortic diameter greater than two is evident

